# Ecological correlates of activity-related behavior typologies among adolescents

**DOI:** 10.1186/s12889-019-7386-9

**Published:** 2019-08-03

**Authors:** Kate E. Parker, Jo Salmon, Karen Villanueva, Suzanne Mavoa, Jenny Veitch, Helen L. Brown, Anna Timperio

**Affiliations:** 1 Institute for Physical Activity and Nutrition (IPAN), Deakin University, Geelong, VIC Australia; 2 Centre for Urban Research, School of Global Urban and Social Studies, RMIT University, Melbourne, Australia; 3 Melbourne School of Population and Global Health, University of Melbourne, Melbourne, Australia

**Keywords:** Typologies, Clustering, Physical activity, Sedentary behavior, Ecological framework, Correlates, Geographic information systems

## Abstract

**Background:**

Adolescents engage in various combinations (typologies) of physical activity and sedentary behaviors, which impact their health and wellbeing in different ways. As such, there is a need to understand the factors that may inhibit or facilitate engagement in combinations of activity-related behaviors to help inform effective intervention strategies targeting those most in need. The aim of this study was to identify ecological correlates of adolescent physical activity and sedentary behavior typologies.

**Methods:**

Cross-sectional study of 473 adolescents (15.0 ± 1.6 years, 41.4% boys) from 18 secondary schools in Melbourne, Australia. Intrapersonal, interpersonal and neighborhood-physical environmental factors were assessed via self-report surveys and Geographic Information Systems. Multinomial logistic regression models determined the relative risk ratio of membership of three homogenous activity-related behavior typologies based on the potential correlates.

**Results:**

Higher levels of self-efficacy for physical activity, parental screen-time restriction rules, parental support for physical activity, sibling screen-time co-participation and perceptions of neighborhood pedestrian/traffic safety were associated with greater likelihood of adolescents being in the typology defined as highly active and low sedentary compared to the physically inactive, highly sedentary typology. Higher frequency of co-participation in screen-time with friends was associated with greater likelihood of adolescents being in the typology defined as moderately active, high screen-time compared to physically inactive, highly sedentary.

**Conclusions:**

A range of intrapersonal, interpersonal and environmental correlates appear to play a role in adolescent activity-related typology membership. The findings may inform public health interventions targeting unique adolescent subgroups most at risk of poor health outcomes based on their engagement in combinations of activity-related behaviors.

**Electronic supplementary material:**

The online version of this article (10.1186/s12889-019-7386-9) contains supplementary material, which is available to authorized users.

## Background

Engagement in physical activity behaviors including sport, active travel and leisure-time active play are positively associated with health and wellbeing [1]. Conversely, sedentary behaviors such as TV viewing, electronic media use, video gaming and sedentary homework are negatively associated with health and wellbeing during adolescence [2]. Despite this, as little as 20–40% of adolescents worldwide achieve the recommended levels of physical activity (≥60 min/day) and sedentary behavior (≤2 h/day of recreational screen-time) [3]. However, adolescents engage in different combinations of activity-related behaviors [4, 5]. Subgroups (typologies) of adolescents engage in beneficial levels of one behavior combined with detrimental levels of the other, beneficial levels of both behaviors, or detrimental levels of both behaviors [4]. Given the clustering of these behaviors and their health implications [6], improving our understanding of activity-related typologies could help inform the development of more effective interventions and target those most in need [4, 5]. An important, but little examined, step required to tailor such interventions is to understand the key correlates of activity-related typologies, or factors that inhibit or facilitate engagement in combinations of activity-related behaviors.

Ecological frameworks have been widely used to help researchers understand and predict the multiple influences on adolescents’ physical activity and sedentary behavior [7]. While many studies have reported intrapersonal, interpersonal and physical environmental correlates of physical activity and sedentary behavior separately, very few studies have sought to identify correlates of typologies, or combinations of these behaviors within an ecological framework. Two studies have assessed ecological correlates of activity-related behavior typologies among adolescents, however, one of these studies included girls only [8] and both included additional behaviors such as socializing, employment and sleep in determining typologies [8, 9] which may influence the correlates identified. Furthermore, while these previous studies explored sociodemographic correlates and a variety of intrapersonal correlates (e.g., perceived sport competence, enjoyment of physical activity and sedentary pursuits and self-efficacy), only a limited number of interpersonal (time with friends, family and friend support) and environmental (household technology density, neighborhood socio-economic status and organizational level barriers) correlates have been assessed [8, 9]. More research is required to examine a wider range of correlates known to be associated with physical activity and sedentary behaviors when these behaviors are examined independently. This should include interpersonal (e.g., co-participation with friends, family and parents [10, 11]) and environmental (e.g., neighborhood walkability, land use, access to neighborhood physical activity destinations and distance to school [12, 13]) correlates known to influence participation in activity-related behaviors. Additionally, no typology studies to date have investigated objectively-assessed aspects of the physical neighborhood environment. For example, greenspace in close proximity to home has been associated with moderate-to vigorous-intensity physical activity (MVPA) [14] and sedentary behavior (weekdays) [15] among youth, and may therefore predict typology membership.

We have previously identified typologies of activity-related behaviors based on self-reported participation in different types of physical activity and sedentary behaviors, and accelerometer-measured MVPA and sedentary time [16]. Three typologies were identified: Typology 1: Physically inactive, highly sedentary; Typology 2: Moderately active, high screen-time; Typology 3: Highly active, low sedentary.

This study aims to explore intrapersonal, interpersonal and physical environmental correlates of these previously identified activity-related behavioral typologies among adolescents. An understanding of the factors that may inhibit or facilitate engagement in combinations of activity-related behaviors, particularly less healthy combinations of physical activity and sedentary behaviors, is needed to tailor interventions appropriately.

## Methods

This study draws data from the NEighbourhood Activity in Youth (NEArbY) Study and conforms to the cross-sectional study reporting STROBE checklist (Additional file 3). Methodological details have been reported elsewhere [16]. In brief, NEArbY was a cross-sectional study with participants recruited across high and low ‘walkable’ areas within high and low income areas of Melbourne, Australia in 2014 and 2015. A total of 528 adolescents from 18 secondary schools provided informed consent to participate in the study, however, due to participant withdrawal and absences on the day of data collection (*n* = 55), the final sample included 473 adolescents (mean age = 15.0 ± 1.6 years, 41.4% boys).

### Activity-related behavior typologies

This study uses typologies of activity-related behaviors previously identified in the NEArbY study and reported descriptively by Parker et al. [16]: Typology 1: Physically inactive, highly sedentary; Typology 2: Moderately active, high screen-time; Typology 3: Highly active, low sedentary. These typologies were based on objectively measured sedentary time (SED), moderate-to-vigorous physical activity (MVPA) and self-reported sport, active transport and specific sedentary behaviors. Briefly, participants were asked to wear an ActiGraph accelerometer (model GT3X+), a valid, reliable and objective measure of their physical activity and sedentary time [17], for eight consecutive days during waking hours. Mean mins/day of SED (≤100 cpm) and MVPA (≥4METs using age-specific cut-points [18]) were dichotomized at the medians. Freedson adult cut-points [19] were applied if aged ≥18 years (*n* = 2). All self-report variables [20] were also dichotomized: participation in sport teams or physical activities outside of school (< 1 vs ≥1 team/activity), active travel to and from school during a typical week (<once vs ≥ once/week) and engagement in TV/DVD/video viewing (< 2 vs ≥2 h/day), video gaming, using electronic media and sedentary homework time (each dichotomized at < 1 vs ≥1 h/day). Latent class analysis was conducted in MPlus (version 7.31) [21] among 473 participants to identify activity-related behavioral typologies based on the accelerometer and self-report data. Class sizes ranging from two to five were tested and the three class solution was found to be optimal based on a number of statistical indicators, as detailed previously [16]. Membership in this three class solution is used here for further analysis.

### Potential correlates

The self-report survey included 15 items assessing intrapersonal, interpersonal and physical environmental factors that have previously been associated with individual activity-related behaviors among adolescents. See Additional file 1: Table S1 for details regarding survey items, source, data reduction and alpha scores for computed scales for the potential correlates included in the analysis.

At the intrapersonal level, items included self-efficacy for physical activity (6 item score; e.g., how sure are you that you can do physical activity even when you feel sad or stressed) [22], enjoyment of sedentary behavior (single item, 5 point likert scale) [23], perceived sport competence (6 item score; e.g., I learn new sport-related skills quickly) adapted from Lindwall et al. [24], and perceived cons (5 item score; e.g., I would feel embarrassed if people saw me doing physical activity) and pros (5 item score; e.g., I would have more energy if I did physical activity) of engaging in physical activity [22].

At the interpersonal level, items included: parental rules restricting screen-time (6 item score; e.g., No TV during meal times) [20], frequency of co-participation in screen-time with parents/guardians, siblings and friends (3 items; never, 1–2 days/week, 3–4 days/week, 5–6 days/week, every day, N/A) [20], parental support for physical activity (5 item score; e.g., Provide transportation to physical activity or sport) [22] and friend/sibling support for physical activity (3 item score; e.g., Do physical activity or play sports with you) [22].

Items assessing the physical environment included the presence of screen based items in the bedroom (sum of 7 items; e.g., TV), physical activity items in the home environment (sum of 10 items; e.g., Bike, basketball hoop) [25], perceived neighborhood pedestrian/traffic safety (8 item score; e.g., I feel safe crossing the streets in my neighborhood) [26] and perceived neighborhood crime safety (7 item score; e.g., There is a high crime rate in my neighborhood) [26].

Spatial data and Geographic Information Systems (GIS) analysis were used to generate objective measures of neighborhood environmental features that may be correlated with activity patterns (see Additional file 2: Table S2 for details of how these neighborhood environmental variables were created). Briefly, participants’ residential addresses were geocoded using cadastral data and linked with GIS data using ArcGIS v10.2 (ESRI, Redlands). The count of private and public recreational land [27–31], the count of trails [32], and the density of parks [30] and public transportation stops [33] within a 1 km walkable road network buffer [32] around home were calculated following established protocols [34].

### Data analysis and data management

Multinomial logistic regression models were used to determine the relative risk of typology membership for each of the potential correlates (‘physically inactive, highly sedentary’ typology as referent group), controlling for sex of the participant and accounting for clustering by school using STATA (version 14). Although significant differences in age were seen between typologies, this was largely school dependent. Age was therefore not added as a confounder as the model already accounted for school attended. Each correlate was entered into separate models to determine unadjusted bivariable relationships. Correlates that were significantly associated with typology membership (*p* < 0.05) in individual models were included in the fully adjusted model.

## Results

The final sample included 473 adolescents (41.4% boys) with a mean age of 15.0 ± 1.6 years. Roughly, three-quarters of participants were of a healthy weight status, lived in neighborhoods of average socio-economic position and were of Australian cultural identity. Approximately one in two owned a dog and reported mostly receiving school grades of As and Bs. One in three had some regular paid employment. Table 1 presents descriptive information for all potential correlates that were assessed for each of the three typologies (Typology 1: Physically inactive, highly sedentary (*n* = 209, 44%); Typology 2: Moderately active, high screen-time (*n* = 198, 42%); Typology 3: Highly active, low sedentary (*n* = 66, 14%)).

**Table 1:** Mean (SD) of activity-related behaviors and potential intrapersonal, interpersonal and physical environmental correlates for each typology

	Overall (*n* = 473)	Typology 1 Physically inactive, highly sedentary (*n* = 209)	Typology 2 Moderately active, high screen-time (*n* = 198)	Typology 3 Highly active, low sedentary (*n* = 66)
Activity-related behaviors				
MVPA mins/day	40.52 (24.66)	33.45 (27.91)	41.28 (20.39)	56.65 (16.43)
SED mins/day	545.97 (90.65)	573.59 (92.73)	545.04 (88.27)	477.89 (43.29)
Potential correlate (range of scores)				
Self-efficacy for physical activity (−12–12)	1.8 (5.9)	0.7 (6.0)	2.1 (5.7)	4.7 (5.2)
Enjoyment of sedentary behaviors (−2–2)	0.9 (1.1)	0.8 (1.2)	1.0 (1.1)	0.8 (1.0)
Sport competence (−12–12)	4.5 (6.5)	3.5 (6.6)	4.6 (6.5)	7.2 (5.3)
Cons of physical activity (−10–10)	−6.3 (3.5)	−5.8 (3.8)	−6.5 (3.2)	−7.2 (2.7)
Pros of physical activity (−10–10)	7.8 (2.5)	7.7 (2.6)	7.8 (2.6)	8.3 (2.0)
Parental screen-time restriction rules (0–6)^1^	1.7 (1.5)	1.5 (1.6)	1.6 (1.4)	2.4 (1.6)
Sibling screen-time co-participation (0–7 days/week)	3.2 (2.0)	2.9 (1.9)	3.3 (2.2)	3.7 (2.0)
Parent/guardian screen-time co-participation (0–7 days/week)	3.0 (2.0)	2.8 (1.9)	3.1 (2.2)	3.3 (1.8)
Friend screen-time co-participation (0–7 days/week)	3.1 (1.7)	2.9 (1.7)	3.3 (1.6)	3.1 (1.6)
Parental support for physical activity (5–25)	16.5 (4.6)	15.4 (5.0)	16.7 (4.2)	19.1 (3.4)
Friend/sibling support for physical activity (3–15)	8.0 (2.9)	7.5 (3.0)	8.2 (2.9)	8.9 (2.5)
Access to screen based items in bedroom (0–7 items)	2.9 (1.6)	2.8 (1.7)	2.9 (1.7)	2.9 (1.4)
Availability of options for physical activity in home environment (0–10 items)	6.1 (2.3)	5.8 (2.3)	6.1 (2.2)	7.1 (2.2)
Perceived neighborhood road safety (−16–16)	4.9 (4.3)	4.5 (4.4)	4.9 (4.3)	6.3 (4.1)
Perceived neighborhood crime safety (−14–14)^2^	−8.6 (5.7)	−8.4 (5.8)	−8.6 (5.7)	−9.6 (5.1)
Count of private/public recreation land (0–16)	1.5 (1.9)	1.4 (1.7)	1.5 (1.7)	1.9 (2.7)
Count of public trails < 1 km from home (0–4)	0.4 (0.6)	0.3 (0.6)	0.4 (0.6)	0.4 (0.7)
Count of public parks < 1 km from home (0–50)	13.3 (9.0)	13.4 (9.5)	13.4 (8.7)	12.9 (8.6)
Density of public transportation < 1 km from home (0–58.7)	25.7 (12.9)	25.5 (13.4)	26.1 (12.4)	25.3 (13.0)

*GIS* Geographic Information Systems, *MVPA* Moderate to-vigorous physical activity, *SED* Sedentary time

Note: Higher scores for potential correlates indicate hypothetically greater support for physical activity or sedentary behaviour, except otherwise indicated; ^1^: Higher score reflects more rules restricting screen-time; ^2^: Higher score reflects higher perceived crime, see Table S1 for further details regarding scoring

Several correlates were significantly associated with typology membership (Table 2). At the intrapersonal level, self-efficacy was the only correlate significantly associated with typology membership within the fully adjusted model (Model 2). For every unit increase in self-efficacy for physical activity, participants were 8% (RR = 1.08) more likely to be classed as ‘highly active, low sedentary’ compared to ‘physically inactive, highly sedentary’. At the interpersonal level, with each unit increase in parental screen-time restriction rules and parental support for physical activity, and each additional day/week of co-participation in screen-time with siblings, participants were more likely to be in the ‘highly active, low sedentary’ typology compared to the ‘physically inactive, highly sedentary’ typology by 32% (RR = 1.32), 15% (RR = 1.15) and 26% (RR = 1.26) respectively. Each additional day/week of co-participation in screen-time with friends was associated with a 14% (RR = 1.14) greater likelihood of adolescents being in the ‘moderately active, high screen-time’ typology compared to the ‘physically inactive, highly sedentary’ typology. Perceived neighborhood pedestrian/traffic safety was the only significant physical environmental correlate. For each unit increase in perceived pedestrian/traffic safety, adolescents were 10% (RR = 1.10) more likely to be in the ‘highly active, low sedentary’ typology compared to the ‘physically inactive, highly sedentary’ typology.

**Table 2:** Relative risk of typology membership according to ecological correlates

	Model 1^a^ RR (95%CI)			Model 2^b^ RR (95%CI)		
	Typology 1 Physically inactive, highly sedentary (Ref)	Typology 2 Moderately active, high screen-time	Typology 3 Highly active, low sedentary	Typology 1 Physically inactive, highly sedentary (Ref)	Typology 2 Moderately active, high screen-time	Typology 3 Highly active, low sedentary
Intrapersonal						
Self-efficacy for physical activity	1.00	1.02 (1.00–1.05)*	1.13 (1.06–1.21)*	1.00	1.03 (0.99–1.06)	1.08 (1.00–1.16)*
Enjoyment of sedentary behaviors	1.00	1.22 (0.95–1.56)	1.02 (0.82–1.27)	1.00	N/A	N/A
Sport competence	1.00	1.01 (0.99–1.05)	1.11 (1.04–1.18)*	1.00	0.98 (0.93–1.03)	1.00 (0.93–1.08)
Cons of physical activity	1.00	0.95 (0.90–1.01)	0.88 (0.81–0.95)*	1.00	0.95 (0.93–1.03)	0.98 (0.87–1.10)
Pros of physical activity	1.00	1.02 (0.93–1.12)	1.12 (1.02–1.24)*	1.00	0.96 (0.85–1.09)	0.98 (0.84–1.15)
Interpersonal						
Screen-time restriction rules	1.00	1.05 (0.97–1.14)	1.41 (1.19–1.67)*	1.00	1.03 (0.92–1.14)	1.32 (1.06–1.64)*
Sibling screen-time co-participation	1.00	1.09 (0.93–1.27)	1.22 (1.08–1.37)*	1.00	1.08 (0.92–1.26)	1.26 (1.08–1.46)*
Parent/guardian screen-time co-participation	1.00	1.07 (0.97–1.19)	1.13 (0.99–1.29)	1.00	N/A	N/A
Friend screen-time co-participation	1.00	1.14 (1.04–1.26)*	1.05 (0.90–1.24)	1.00	1.14 (1.00–1.30)*	1.01 (0.85–1.20)
Parental support for physical activity	1.00	1.06 (1.01–1.12)*	1.23 (1.15–1.31)*	1.00	1.03 (0.96–1.11)	1.15 (1.03–1.28)*
Friend/sibling support for physical activity	1.00	1.10 (1.02–1.18)*	1.19 (1.08–1.32)*	1.00	1.06 (0.97–1.17)	1.04 (0.87–1.24)
Physical environmental						
Access to screen based items in bedroom	1.00	1.03 (0.92–1.17)	1.03 (0.85–1.24)	1.00	N/A	N/A
Availability of options for physical activity in home environment	1.00	1.06 (0.96–1.16)	1.31 (1.16–1.47)*	1.00	1.04 (0.94–1.14)	1.12 (0.95–1.33)
Perceived neighborhood road safety	1.00	1.01 (0.97–1.06)	1.10 (1.05–1.16)*	1.00	1.02 (0.95–1.09)	1.10 (1.01–1.18)*
Perceived neighborhood crime safety	1.00	1.02 (0.99–1.05)	0.97 (0.92–1.02)	1.00	N/A	N/A
GIS: Count of private/public recreation land	1.00	1.06 (0.97–1.17)	1.15 (0.98–1.35)	1.00	N/A	N/A
GIS: Count of public trails	1.00	1.20 (0.84–1.72)	1.07 (0.66–1.76)	1.00	N/A	N/A
GIS: Count of public parks	1.00	0.99 (0.98–1.01)	0.99 (0.96–1.02)	1.00	N/A	N/A
GIS: Density of public transportation	1.00	1.01 (0.99–1.02)	1.00 (0.97–1.03)	1.00	N/A	N/A

^a^Adjusted for sex and accounting for clustering by school; ^b^Additionally adjusted for all variables significant in unadjusted analyses; *p < 0.05; NA – was not included in the model due to non-significant results in the unadjusted model; GIS: Geographic Information Systems

## Discussion

This study examined the intrapersonal, interpersonal and physical environmental correlates associated with relative risk of membership in three unique activity-related behavioral typologies. Interpersonal level factors (screen-time restriction rules, friend and sibling co-participation in screen-time and parental support for physical activity) were more commonly associated with typology membership among this sample of adolescents than intrapersonal and physical environmental factors. Just one intrapersonal factor (self-efficacy) and one physical environmental factor (perceived neighborhood pedestrian/traffic safety) predicted typology membership.

The positive association between self-efficacy for physical activity and the likelihood of adolescents being in the high physical activity, low sedentary typology relative to low physical activity and high sedentary behavior typology is consistent with previous research around the role that self-efficacy plays in physical activity participation [35], irrespective of sedentary behavior engagement. Previous typology studies that focused solely on adolescent physical activity and sedentary behaviors have not assessed the influence of self-efficacy for physical activity on typology membership. However, a time-use study found that adolescent girls who were defined as being ‘highly involved in all daily activities’ were more likely to self-report high self-efficacy for physical activity when compared to those defined as ‘screenies’ [8]. Similarly, research that identified typologies based on physical activity, sedentary behavior and health risk-related behaviors (such as alcohol use and smoking) found that adolescents with higher self-efficacy for physical activity were less likely to be classified as sedentary and engaging in high risk behaviors compared to those classified as having healthy physical activity levels and low risk health behaviors [36]. Self-efficacy for reducing sedentary behavior has yet to be assessed within studies of adolescent activity-related typologies, highlighting a gap for future research.

The results in regards to parental screen-time restriction rules support previous findings from Zabinski et al. [37] who identified clusters of sedentary behaviors among adolescents and found that adolescents with parents who enforced rules around limiting TV and computer use were more likely to be in a cluster defined by low levels of sedentary behavior. However, no previous typology studies comprising both physical activity and sedentary behaviors have assessed screen-time rules. Therefore more research is needed to establish consistency in findings and future studies should also assess whether enforcing rules for physical activity (e.g., must be active for an hour a day) also influences engagement in these activity-related behavior combinations.

Perceived parental support for physical activity, including both practical and emotional support, was associated with a higher likelihood of being in the typology defined by high physical activity, low sedentary relative to low physical activity, high sedentary behavior. Consistent with previous physical activity research with children and adolescents [38], the findings highlight that adolescents still rely on support from parents at this age. While no previous typology studies have explored sibling or friend screen-time co-participation, studies have suggested that siblings and friends play a role in both physical activity and sedentary behavior independently. In this study, higher frequency of sibling screen-time co-participation was associated with a greater likelihood of being in the typology characterized by high physical activity, low sedentary compared to low physical activity, high sedentary. These findings are counterintuitive; however, it is possible that the classification of high physical activity indicates that siblings are engaging in active gaming such as Nintendo Wii rather than more sedentary screen time. Further research is needed to explore this association. In comparison, higher frequency of friend screen-time co-participation was associated with a greater likelihood of being in the moderate activity, high screen-time typology relative to the low physical activity, high sedentary typology. It is likely that friendships evolve as a result of similarities in behavior preferences, however due to the cross-sectional nature of the study it is difficult to determine the nature of this relationship.

Overall, the findings suggest that family and friends are key factors related to adolescents’ engagement in activity-related typologies, particularly screen-based sedentary time, and should be targeted in future intervention strategies. To date, family and community-based interventions designed to decrease sedentary time have focused predominantly on parental support, rule setting and integration of technology (e.g., active video gaming or electronic timers) and have been moderately successful [39]. Fewer interventions have included strategies targeting friends and siblings, which according to our findings may help reduce screen-time and overall sedentary time in conjunction with increasing physical activity.

Although none of the objectively measured neighborhood environment features were associated with typology membership, perceived greater neighborhood pedestrian/traffic safety was associated with a lower risk of being in the low physical activity, high sedentary typology compared to the high physical activity, low sedentary typology. While difficult to make direct comparisons due to no other typology studies assessing these factors, this contradicts findings from a 2007 review of environmental correlates in youth which found no association between perceived neighborhood safety and physical activity levels among adolescents [40]. Conversely, objectively measured neighborhood environment features have been found to be associated with physical activity engagement including an inverse relationship with crime incidence [40], and cul-de-sac density and proportion of low speed limit streets among adolescent girls [41]. It is important to acknowledge that the current study only assessed objective neighborhood environmental features within a 1 km buffer of participants’ home. Adolescents in Australia do not necessarily live in close proximity to their school, and it is possible that the school environment also plays an important role in combined activity levels during the adolescent years. Future research should examine a larger buffer around participants’ homes, as well as associations between the physical environmental features around school and behavioral typology membership, particularly objective measures of traffic safety and volume.

This study was cross-sectional, therefore results cannot infer a directional or causal relationship. Future research into the correlates of activity-related behavioral typologies should assess how these correlates predict change or stability in typology membership over time. Strengths of this study included the diversity of participants across high and low ‘walkable’ areas within high and low-income areas, and objective measures of physical activity, sedentary behaviors and neighborhood environmental features. Additionally, this study included a greater range of potential correlates than previous typology studies; however, most of the correlates related to physical activity (e.g., physical activity self-efficacy). Future research should include a greater range of explanatory variables focused on sedentary behavior. Additionally, to allow for an understanding of influences on the entire continuum of activity, light-intensity activities should also be considered within typology composition.

## Conclusions

Overall, the findings suggest that self-efficacy for physical activity, parental rules and support, sibling and friend co-participation and perceptions of neighborhood road safety may have a significant influence on co-occurring activity-related behaviors during adolescence. These correlates stem from multiple levels of the ecological framework and may be useful to help inform public health interventions targeting unique adolescent subgroups most at risk of poor health outcomes based on their activity-related behavior combinations.

## Additional files

Additional file 1: **Table S1.** Potential ecological correlates of activity-related health behaviors. This table provides detail regarding variables that were assessed as potential correlates of activity-related health behaviors including the survey items used and data reduction for use of the variables. (DOCX 26 kb)

Additional file 2: **Table S2.** GIS data sources and calculations for neighborhood environmental features. This table provides detail regarding the geographical information systems data sources and the calculations performed to determine objective neighborhood environmental features to be assessed. (DOCX 15 kb)

Additional file 3: STROBE checklist for cross-sectional studies. This file provides the STROBE checklist for this manuscript. (DOC 88 kb)

## Data Availability

The datasets generated and/or analyzed during the current study are not publicly available due to ethical restrictions related to participant consent obtained at the commencement of the study. An ethically compliant dataset may be available from the corresponding author on reasonable request. Please contact hos_ens@deakin.edu.au with any data requests.

## Abbreviations

GIS: Geographic Information Systems
MVPA: Moderate-to vigorous-intensity physical activity
RRR: Relative Risk Ratio

## References

[1] Granger E, Di Nardo F, Harrison A, Patterson L, Holmes R, Verma A (2017). A systematic review of the relationship of physical activity and health status in adolescents. Eur J Pub Health.

[2] Carson V, Hunter S, Kuzik N, Gray CE, Poitras VJ, Chaput J-P, Saunders TJ, Katzmarzyk PT, Okely AD, Connor Gorber S (2016). Systematic review of sedentary behaviour and health indicators in school-aged children and youth: an update. Appl Physiol Nutr Metab.

[3] Tremblay MS, Barnes JD, González SA, Katzmarzyk PT, Onywera VO, Reilly JJ, Tomkinson GR (2016). Global matrix 2.0: report card grades on the physical activity of children and youth comparing 38 countries. J Phys Act Health.

[4] Ferrar K, Chang C, Li M, Olds TS (2013). Adolescent time use clusters: a systematic review. J Adolesc Health.

[5] Leech RM, McNaughton SA, Timperio A (2014). The clustering of diet, physical activity and sedentary behavior in children and adolescents: a review. Int J Behav Nutr Phys Act.

[6] Lee PH (2014). Association between Adolescents’ physical activity and sedentary behaviors with change in BMI and risk of type 2 diabetes. PLoS One.

[7] Sallis JF, Owen N, Fisher EB, Glanz K, Rimer BK, Viswanath K, Glanz K, Rimer BK, Viswanath K (2008). Ecological models of health behavior. Health behavior and health education: Theory, research, and practice (4th ed). Edn.

[8] Casey M, Harvey J, Telford A, Eime R, Mooney A, Payne W (2016). Patterns of time use among regional and rural adolescent girls: associations with correlates of physical activity and health-related quality of life. J Sci Med Sport.

[9] Gorely T, Marshall SJ, Biddle SJH, Cameron N (2007). Patterns of sedentary behaviour and physical activity among adolescents in the United Kingdom: project STIL. J Behav Med.

[10] Minges KE, Owen N, Salmon J, Chao A, Dunstan DW, Whittemore R (2015). Reducing youth screen time: qualitative Metasynthesis of findings on barriers and facilitators. Health Psychol.

[11] Limstrand T (2008). Environmental characteristics relevant to young people's use of sports facilities: a review. Scand J Med Sci Sports.

[12] Davison KK, Lawson CT (2006). Do attributes in the physical environment influence children's physical activity? A review of the literature. Int J Behav Nutr Phys Act.

[13] Sirard JR, Slater ME (2008). Walking and bicycling to school: a review. Am J Lifestyle Med.

[14] McCrorie PRW, Fenton C, Ellaway A (2014). Combining GPS, GIS, and accelerometry to explore the physical activity and environment relationship in children and young people - a review. Int J Behav Nutr Phys Act.

[15] Bringolf-Isler Bettina, de Hoogh Kees, Schindler Christian, Kayser Bengt, Suggs L., Dössegger Alain, Probst-Hensch Nicole (2018). Sedentary Behaviour in Swiss Children and Adolescents: Disentangling Associations with the Perceived and Objectively Measured Environment. International Journal of Environmental Research and Public Health.

[16] Parker KE, Salmon J, Brown HL, Villanueva K, Timperio A (2019). Typologies of adolescent activity related health behaviours. J Sci Med Sport.

[17] Montoye AH, Pfeiffer KA, Suton D, Trost SG (2014). Evaluating the responsiveness of accelerometry to detect change in physical activity. Meas Phys Educ Exerc Sci.

[18] Trost SG, Pate RR, Sallis JF, Freedson PS, Taylor WC, Dowda M, Sirard J (2002). Age and gender differences in objectively measured physical activity in youth. Med Sci Sports Exerc.

[19] Freedson PS, Melanson E, Sirard J (1998). Calibration of the computer science and applications, Inc. accelerometer. Med Sci Sports Exerc.

[20] Active Where? Individual item reliability statistics adolescent survey. [http://activelivingresearch.org/sites/default/files/AW_item_reliability_Adolescent.pdf]. Accessed 10 Aug 2018.

[21] Muthen L, Muthen B (2015). Mplus users guide, seventh edition edn.

[22] Norman GJ, Sallis JF, Gaskins R (2005). Comparability and reliability of paper- and computer-based measures of psychosocial constructs for adolescent physical activity and sedentary behaviors. Res Q Exerc Sport.

[23] Norman GJ, Schmid BA, Sallis JF, Calfas KJ, Patrick K (2005). Psychosocial and environmental correlates of adolescent sedentary behaviors. Pediatrics.

[24] Lindwall M, Asci H, Hagger MS (2011). Factorial validity and measurement invariance of the revised physical self-perception profile (PSPP-R) in three countries. Psychol Health Med.

[25] Rosenberg DE, Sallis JF, Kerr J, Maher J, Norman GJ, Durant N, Harris SK, Saelens BE (2010). Brief scales to assess physical activity and sedentary equipment in the home. Int J Behav Nutr Phys Act.

[26] Rosenberg D, Ding D, Sallis JF, Kerr J, Norman GJ, Durant N, Harris SK, Saelens BE (2009). Neighborhood environment walkability scale for youth (NEWS-Y): reliability and relationship with physical activity. Prev Med.

[27] State Government of Victoria: Vicmap property (2013). Environment DoSa.

[28] Pitney Bowes Ltd (2014). Axiom business points.

[29] State Government of Victoria: VGO data (2010). Environment DoSa.

[30] State Government of Victoria (2011). Public Open Space Inventory. Council VEA.

[31] Sports Victoria (2015). Sports Facilities.

[32] State Government of Victoria: Vicmap Transport (2013). Environment DoSa.

[33] State Government of Victoria (2015). Public Transport Stops (General Transit Feed Specification). Victoria PT.

[34] Adams MA, Chapman JC, Sallis JF, Frank LD (2015). The International Physical Activity and Environment Network (IPEN) Study Coordinating Center: Built Environment and Physical Activity: GIS Templates and Variable Naming Conventions, Version 2. For the IPEN Adolescent Study.

[35] Sterdt E, Liersch S, Walter U (2014). Correlates of physical activity of children and adolescents: a systematic review of reviews. Health Educ J.

[36] Busch V, Van Stel HF, Schrijvers AJP, de Leeuw JRJ (2013). Clustering of health-related behaviors, health outcomes and demographics in Dutch adolescents: a cross-sectional study. BMC Public Health.

[37] Zabinski MF, Norman GJ, Sallis JF, Calfas KJ, Patrick K (2007). Patterns of sedentary behavior among adolescents. Health Psychol.

[38] Ha A, Abbott R, Macdonald D, Pang B (2009). Comparison of perceived support for physical activity and physical activity related practices of children and young adolescents in Hong Kong and Australia. Eur Phys Educ Rev.

[39] Marsh S, Foley LS, Wilks DC, Maddison R (2014). Family-based interventions for reducing sedentary time in youth: a systematic review of randomized controlled trials. Obes Rev.

[40] Ferreira I, van der Horst K, Wendel-Vos W, Kremers S, van Lenthe FJ, Brug J (2007). Environmental correlates of physical activity in youth – a review and update. Obes Rev.

[41] van Loon J, Frank LD, Nettlefold L, Naylor P-J (2014). Youth physical activity and the neighbourhood environment: examining correlates and the role of neighbourhood definition. Soc Sci Med.

