# Supplementary material for: Ecological correlates of activity-related behavior typologies among adolescents
Source: BMC Public Health. 2019 Aug 3;19:1041. doi: 10.1186/s12889-019-7386-9 (PMC6679435; doi:10.1186/s12889-019-7386-9)
Supplement: Supplementary file 1 — Table S1. Potential ecological correlates of activity-related health behaviors. This table provides detail regarding variables that were assessed as potential correlates of activity-related health behaviors including the survey items used and data reduction for use of the variables. (DOCX 26 kb) [file 12889_2019_7386_MOESM1_ESM.docx]

Table S1. Potential ecological correlates of activity-related health behaviours

| **Potential correlate** | **Survey items** | **Data reduction** | **Alpha** |
| --- | --- | --- | --- |
| **Intrapersonal** | | | |
| Self-efficacy for physical activity^a^[22] | How sure are you that you can?...   1. Do physical activity even when you feel sad or stressed 2. Set aside time for physical activity on most days of the week 3. Do physical activity even when your family or friends want you to do something else 4. Get up early, even on weekends, to do physical activity 5. Do physical activity even when you have a lot of homework 6. Do physical activity even when it is raining or really hot outside | -2 = I’m sure I can’t  -1 = 2  0 = 3  1 = 4  2 = I’m sure I can  Self-efficacy score = sum of all 6 question responses | 0.84 |
| Enjoyment of sedentary behaviour ^a^[23] | I enjoy doing sedentary activities like watching TV, playing on the computer/video games or using the internet | -2 = Strongly disagree  -1 = Somewhat disagree  0 = Neutral  1 = Somewhat agree  2 = Strongly agree | N/A |
| Sport competence[24] | How much do you agree/disagree?   1. I feel confident when playing most sports 2. I am always among the first to join in sports activities 3. I learn new sport-related skills quickly 4. I am very good at playing sports 5. I consider myself as an athlete/sporty person 6. I am always willing to join in on sport activities | -2 = Strongly disagree  -1 = Somewhat disagree  1 = Somewhat agree  2 = Strongly agree  Sport competence score = sum of all 6 question responses | 0.92 |
| Perceived cons of physical activity^a^ [22] | How much do you agree/disagree?   1. I would feel embarrassed if people saw me doing physical activity 2. There is too much I would have to learn to do physical activity 3. I would need too much help from my parents to do physical activity 4. I do not like the way physical activity and exercise makes me feel 5. Physical activity takes time away from being with my friends | -2 = Strongly disagree  -1 = Somewhat disagree  1 = Somewhat agree  2 = Strongly agree  Physical activity cons score = sum of all 5 question responses | 0.60 |
| Perceived pros of physical activity ^a^[22] | How much do you agree/disagree?   1. Physical activity would help me stay fit 2. My parents would be happy if I did physical activity 3. I would feel better about myself if I did physical activity 4. I would have fun doing physical activity or playing sports with my friends 5. I would have more energy if I did physical activity | -2 = Strongly disagree  -1 = Somewhat disagree  1 = Somewhat agree  2 = Strongly agree  Physical activity pros score = sum of all 5 question responses | 0.63 |
| **Interpersonal** | | | |
| Screen time restriction rules ^a^[20] | Does your parent or guardian have the following rules?   1. No TV/DVD/computer before homework 2. Less than 2 hours TV/DVD/computer per day 3. No internet use without permission 4. No TV during meal times 5. Limits on internet use 6. No electronic device after a certain time at night | 1 = yes  0 = no  Screen time restriction rules score = sum of all 6 responses | N/A |
| Co-participation of screen time ^a^[22] | During a typical week, how often do you sit and watch TV or play electronic games with…   1. Brothers/sisters 2. A parent/guardian/caregiver 3. Friends | 0 = Never  1.5 = 1-2 days  3.5 = 3-4 days  5.5 = 5-6 days  7 = Every day  0 = N/A  Score each co-participant separately | N/A |
| Parental/adult support for physical activity ^a^[22] | During a typical week, how often does an adult in your household…   1. Encourage you to do sports or physical activity 2. Provide transportation to a place where you can do physical activity or play sports 3. Do physical activity or play sports with you 4. Pay for sporting club fees 5. Discourage your from sitting too much | 1 = Never  2 = Rarely  3 = Sometimes  4 = Often  5 = Very often  Adult support for physical activity score = sum of all 5 responses | 0.74 |
| Friend/sibling support for physical activity ^a^[22] | During a typical week, how often do your brothers/sisters or friends…   1. Do physical activity or play sports with you 2. Ask you to walk or cycle to school or to a friend’s house 3. Discourage you from sitting too much | 1 = Never  2 = Rarely  3 = Sometimes  4 = Often  5 = Very often  Friend/sibling support for physical activity = sum of all 3 responses | 0.65 |
| **Physical environmental** | | | |
| Sedentary behaviour items in bedroom | Indicate whether the following is in your bedroom.   1. TV 2. DVD player 3. Music player 4. Computer 5. Video game system 6. Active video games 7. Internet access | 1 = yes  0 = no  Access to sedentary behaviour in bedroom score = sum of all 7 responses | N/A |
| Physical activity items at home ^a^[20] | How often do you use these items in or around your home?   1. Bike 2. Basketball hoop 3. Jump/skipping rope 4. Active video games 5. Sports equipment 6. Swimming pool 7. Skateboard/scooter/rollerblades 8. Home aerobic equipment 9. Weightlifting equipment 10. Water or snow equipment | 0 = Not available (don’t have)  1 = Available but never use  1 = Once a month or less  1 = Once every other week  1 = Once a week or more  Availability of physical activity options = sum of all 10 items | 0.67 |
| Neighbourhood pedestrian/traffic safety^a^ [26] | Select the answer that best applies to your neighbourhood.   1. There is so much traffic along nearby streets that it makes it difficult or unpleasant to walk in my neighbourhood 2. The speed of traffic on most nearby streets is usually slow 3. Most drivers go faster than the posted speed limits in my neighbourhood 4. When walking in my neighbourhood there are a lot of exhaust fumes 5. My neighbourhood streets have good lighting at night 6. Walkers and cyclist on the streets in my neighbourhood can be easily seen by people in their homes 7. There are pedestrian crossings and traffic lights to help walkers cross busy streets in my neighbourhood 8. I feel safe crossing the streets in my neighbourhood | -2 = Strongly disagree  -1 = Somewhat disagree  1 = Somewhat agree  2 = Strongly agree  Neighbourhood road safety score = sum of all 8 responses | 0.52 |
| Neighbourhood crime safety^a^ [26] | Select the answer that best applies to your neighbourhood.   1. There is a high crime rate in my neighbourhood 2. The crime rate in my neighbourhood makes it unsafe to go on walks alone or with someone at night 3. I am worried about being outside alone around my house because I am afraid of being taken or hurt by a stranger 4. I am worried about being outside with a friend around my home because I am afraid of being taken or hurt by a stranger 5. I am worried about being or walking alone or with friends in my neighbourhood and local streets because I am afraid of being taken or hurt by a stranger 6. I am worried about being in a local/nearby park because I am afraid of being taken or hurt by a stranger 7. I am not allowed in a local/nearby park because my parents are afraid that I might be taken or hurt by a stranger | -2 = Strongly disagree  -1 = Somewhat disagree  1 = Somewhat agree  2 = Strongly agree  Neighbourhood crime safety score = sum of all 7 responses | 0.84 |
| Note: ^a^indicates item is reliable |  |  |  |
