# Supplementary material for: Ecological correlates of activity-related behavior typologies among adolescents
Source: BMC Public Health. 2019 Aug 3;19:1041. doi: 10.1186/s12889-019-7386-9 (PMC6679435; doi:10.1186/s12889-019-7386-9)
Supplement: Supplementary file 2 — Table S2. GIS data sources and calculations for neighborhood environmental features. This table provides detail regarding the geographical information systems data sources and the calculations performed to determine objective neighborhood environmental features to be assessed. (DOCX 15 kb) [file 12889_2019_7386_MOESM2_ESM.docx]

Table S2. GIS data sources and calculations for neighborhood environmental features

| **Neighbourhood environmental variable** | **Calculation details** | **Data sources** |
| --- | --- | --- |
| 1 km walkable road network buffer (i.e. the representation of individual participant’s neighbourhood boundaries) | Road centrelines were extracted from VicMap Transport (2013) and pre-processed to remove non-walkable segments (freeways, freeway on/off-ramps). ArcGIS Network analyst was used to select all walkable road centrelines within 1 km of the participant address. A sausage (or balloon) buffer was calculated by buffering the selected road centrelines by 25 meters. | Road centrelines - State Government of Victoria: Vicmap Transport. Environment DoSa ed.; 2013.[35] |
| Count of private and public recreational land | A range of data sources were used to identify private and public recreational land parcels in the study area. The spatial join function in ArcGIS 10.2 was applied to calculate the number of private and public recreational parcels within each walkable road network buffer. | Parcel boundaries - State Government of Victoria: Vicmap Property. Environment DoSa ed.; 2013.[36]  Land use - Pitney Bowes Ltd, Axiom business points, Pitney Bowes Ltd, Editor. 2014: Sydney.[37]  Land use - State Government of Victoria: VGO data. Environment DoSa ed.; 2010 [38]  Public open spaces - State Government of Victoria: Public Open Space Inventory. Victorian Environmental Assessment Council; 2011 [39]  Sports facilities - Sports Victoria (2015) Sports facilities. [40] |
| Count of trails | Trail data was extracted from VicMap Transport (2013). The spatial join function in ArcGIS 10.2 was applied to calculate the number of trails that intersected each walkable road network buffer. | State Government of Victoria: Vicmap Transport. Environment DoSa ed.; 2013. [35] |
| Density of parks | Parks were extracted from the VEAC Public Open Space Inventory. The spatial join function in ArcGIS 10.2 was applied to calculate the number of parks that intersected each walkable road network buffer. | State Government of Victoria: Public Open Space Inventory. Victorian Environmental Assessment Council; 2011 [39] |
| Density of public transport stops | The spatial join function in ArcGIS 10.2 was applied to calculate the number of public transport stops within each walkable road network buffer. | State Government of Victoria: Public Transport Stops (General Transit Feed Specification). Victoria PT ed.; 2015. [41] |
